# The Association between Diet and Sleep Quality among Spanish University Students

**DOI:** 10.3390/nu14163291

**Published:** 2022-08-11

**Authors:** Enrique Ramón-Arbués, José-Manuel Granada-López, Blanca Martínez-Abadía, Emmanuel Echániz-Serrano, Isabel Antón-Solanas, Benjamin Adam Jerue

**Affiliations:** 1 Faculty of Health Sciences, Campus Universitario Villanueva de Gállego, Universidad San Jorge, Villanueva de Gállego, 50830 Zaragoza, Spain; 2 Research Group Cultural Transferences and International Projection of Aragonese Culture (H27_20D-TRANSFERCULT), 50009 Zaragoza, Spain; 3 Faculty of Health Sciences, Department of Physiatry and Nursing, Universidad de Zaragoza, 50009 Zaragoza, Spain; 4 GIISA021—Seguridad y Cuidados Research Group, 50018 Zaragoza, Spain; 5 Occupational Health and Prevention Service of the Zaragoza City Council, 50001 Zaragoza, Spain; 6 GIIS094—Grupo Enfermero de Investigación en Atención Primaria de Aragón (GENIAPA), 50009 Zaragoza, Spain; 7 Faculty of Communication and Social Sciences, Campus Universitario Villanueva de Gállego, Universidad San Jorge, Villanueva de Gállego, 50830 Zaragoza, Spain

**Keywords:** sleep quality, diet, university students, cross-sectional studies

## Abstract

While it has long been recognized that diet is a leading behavioral risk factor for human health, recent scientific findings have also suggested that diet and sleep quality may be connected. The purpose of the present study is to evaluate the association between diet and sleep quality among a group of Spanish university students. To do so, a cross-sectional study of 868 students was carried out. Sleep quality was assessed using the Spanish version of the Pittsburgh Sleep Quality Index (PSQI), while diet was assessed using the Spanish Healthy Eating Index (SHEI). The study revealed a noteworthy rate of bad sleepers (51.6%) and students whose diet needed modifications (82.2%). Unhealthy eaters were more likely to have poor sleep quality (aOR = 4.20; CI 95%: 2.07–8.52). The unbalanced intake of vegetables (aOR = 1.63; CI 95%: 1.14–2.34), fruits (aOR = 4.08; CI 95%: 2.90–5.74), dairy products (aOR = 1.96; CI 95%: 1.41–2.72), lean meats (aOR = 1.82; CI 95%: 1.19–2.78), legumes (aOR = 1.43; CI 95%: 1.00–2.02), sweets (aOR = 1.60; CI 95%: 1.13–2.25) and sugary soft drinks (aOR = 1.46; CI 95%: 1.07–1.99) was associated with lower sleep quality.

## 1. Introduction

As an essential and complex biological process, sleep can influence a range of physiological functions, such as cerebral activity; metabolism; appetite regulation; and the functioning of the immune, hormone, and cardiovascular systems [1]. Several types of sleeping disorders have been identified that can be characterized by irregularities in the quantity, quality and duration of sleep [2]. In the developed and western worlds, it appears that around 15% [3,4] of the general population suffers from some type of sleeping disorder, though it is probable that such disorders have been underdiagnosed and hence the true number is higher [5].

Sleep disorders have been recognized as risk factors for declines in physical and mental health (both in the long and short terms) and even death [6,7,8]. Hence, those suffering from sleeping problems can experience a general decline in quality of life [9,10] and have a higher mortality rate than the general population [11,12]. Although the precise mechanisms that regulate the relation between sleep quality (SQ) and health have yet to be fully elucidated, it appears quite likely that there are various mechanisms at play involving changes in hormonal levels, metabolic deterioration and inflammatory processes [13,14].

Given the likely influence of SQ on various aspects of health, it is of great important to study the factors that are related to the onset of sleep disorders. Researchers have put forth various etiological and physiopathological models for understanding sleep problems, most of which are implicitly or explicitly based on the “3P” model of Spielman et al. [15], which argues that predisposing, precipitating and perpetuating factors all play a role in sleep disorders; medical conditions (e.g., endocrine, neurological or respiratory problems), psychiatric conditions (e.g., depression, anxiety or psychotropic medications), environmental conditions (e.g., work shifts, the physical conditions of a bedroom or sleep interruptions) and behavioral conditions can all affect sleep on these three different levels [16]. Among the identified behavioral factors, we must include any actions that can negatively impact sleep (e.g., carrying out non-sleep-related activities in a bedroom, the tendency to lie awake in bed or spending excessive amounts of time in bed) [17,18]. More indirectly, other health-related behaviors have also been associated with changes in sleep patterns. These include the intake of substances such as alcohol [19,20] or tobacco [21,22], leading a sedentary lifestyle [23,24] or an excess of screen time [25,26], all of which have been shown to alter sleep homeostasis. Likewise, research over the past decades has highlighted how diet is a changeable lifestyle choice that can also influence sleep patterns. From a perspective focused on macronutrients, the excessive intake of saturated fats, and sugar, alongside low levels of fiber intake, appears to be associated with less restorative sleep [27]. Likewise, the insufficient intake of proteins and carbohydrates could also lead to a decreased duration of sleep [28]. Inversely, there is also evidence suggesting that the intake of certain foods can positively affect sleep. To give a single example, the consumption of bread, legumes, fatty fish and shellfish [29] could increase sleep duration. Likewise, eating nuts [30], dairy [31,32] and some fruits, such as cherries or kiwis [33], may be beneficial for sleep.

Most research focused on the link between diet and sleep quality has been focused on middle-aged or elderly adults. As a result, there has been less research focused on the relationship between sleep and nutrition among young adults and university students in particular. Yet, these young adults find themselves in a crucial stage for the acquisition and consolidation of all types of habits associated with health, including dietary and sleep-related behaviors. There are many variables linked to university life that could lead to specific changes in sleep and dietary habits, including the following: competition between classmates, academic performance, the increased use of screens, time management, the immediate environment (e.g., access to affordable food), or relationships with peers and their influence on behavior [34,35,36]. Given the limited research on the relationship between the sleep and diet quality (DQ) of university students, it is necessary to carry out new, well-designed studies with ample sample sizes. With this objective in mind, we have studied the link between DQ and SQ among a group of Spanish university students.

## 2. Materials and Methods

### 2.1. Design and Study Population

**Figure 1:**
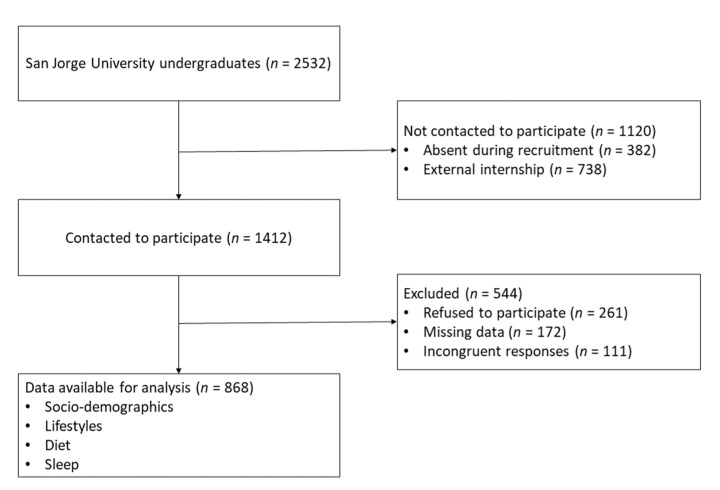
Participant selection flowchart.

A cross-sectional study was carried out that asked participants to fill out questionnaires. The sample included undergraduate students enrolled at the Universidad San Jorge, Spain. In classes during the second semester of the 2020–2021 academic year (i.e., from February to May), students were informed about the study’s objectives and participants were recruited. In total, 1.412 of the 2.532 students enrolled in the university were invited to participate in the study; 261 students declined to do so, while the responses of an additional 283 had to be excluded from the analysis due to incomplete and/or incongruent responses (Figure 1).

### 2.2. Data Collection

The students who agreed to participate were asked to fill out an online questionnaire in the classroom that was designed to maintain anonymity. The questionnaire collected information about sociodemographics (age, gender, place of residence, relationship status and economic status), academics (grade point average) and anthropometrics (height and weight used to calculate BMI), as well as information concerning lifestyle choices (smoking habits, alcohol intake, physical activity, screen time, and diet) and sleep habits.

Sociodemographic, academic and anthropometric data, as well as daily screen time, were self-reported by answering a series of questions written specifically for collecting information for the present study. The CAGE questionnaire was used to assess alcohol consumption. This tool, which has been designed to detect alcohol abuse and dependence, has received an average sensitivity value of 0.71 and an average specificity value of 0.90 [37]. For studying the Spanish population, this questionnaire was validated by Rodríguez Martos et al. (1986) [38]. The CAGE consists of four items that can be answered either affirmatively or negatively. Alcohol consumption is deemed problematic when two or more questions are answered affirmatively. 

Physical activity was assessed using the short version of the international physical activity questionnaire (IPAQ-SF). This tool, which has been validated for studying Spanish university students [39], measures the intensity, frequency and duration of physical activity over the last seven days. The IPAQ-SF allows participants’ activity to be assessed in two ways: first, it offers a calculation of the metabolic equivalent of tasks (METs) taking type of activity (walking, moderate physical activity and vigorous physical activity) and time spent into account; secondly, the tool allows researchers to classify individuals into three categories of physical activity (low, moderate and high) [40].

Information concerning students’ DQ was collected using the Spanish Healthy Eating Index (SHEI) [40], which is an adapted version of Kennedy’s Healthy Eating Index that was designed to better study the Spanish population [41]. The SHEI assesses DQ by asking about 10 different areas of diet: bread and grains, vegetables, fruits, dairy products, meat (including eggs), legumes, cold meats and cuts, sweets, soft drinks with sugar, and diet variety. Each of these sections is scored between 0 and 10 points depending on the degree to which a participant adheres to the Spanish Society of Community Health’s (SENC) recommendations about how often certain foods should be ingested [42]. The criteria used to score each of the 10 SHEI components are detailed in Supplementary Table S1. Once the 10 section scores are added together, final scores can range between 0 and 100 points. Final scores are then used to classify DQ into the following three categories: unhealthy (<51 points), needing modification (51–80 points) and healthy (>80 points) [43].

The Spanish version of the Pittsburgh Sleep Quality Index (PSQI) was used to evaluate participants’ SQ [44]. The PSQI is a self-rated questionnaire that assesses sleep quality and disturbances over a 1-month time interval. This instrument consists of 19 items, which are used to generate 7 subscales: subjective sleep quality, sleep duration, sleep latency, habitual sleep efficiency, use of sleep medications, sleep disturbances, and daytime dysfunction. Each subscale has a range of possible scores from 0 (very good) to 3 (very bad). The sum of these subscales scores yields the global PSQI score (ranging from 0 to 21), with scores above 5 reflecting poor sleep quality and pathological difficulties. This questionnaire has been validated for studying Spanish youth and adolescents and demonstrates good convergent and divergent validity and reliability (Cronbach’s alpha = 0.72) [45].

### 2.3. Data Analysis

The characteristics of the sample have been summarized using mean and standard deviation for the continuous variables and the frequency and percentage for the nominal ones. The Mann–Whitney U tests were employed to compare the characteristics of participants who reported good versus poor sleep quality for quantitative variables, whereas a Chi^2^ test was used for nominal variables. The results for each of the PSQI domains in relation to DQ were compared using the Kruskal–Wallis test and post-hoc pairwise comparisons. Subsequently, different binary logistic regression models (*Enter* method) were run to determine the strength of the correlation between DQ and poor sleep quality. The statistical analysis of the data was performed with the SPSS statistical package for Windows-Version 21 (IBM Corp, Armonk, NY, USA) accepting a significance level of *p* < 0.05.

## 3. Results

In total, the data from 868 university students were analyzed for the study. The average age of the sample was 22.84 ± 7.50, and participants were overwhelmingly female (78.2%). Most participants were not smokers (67.7%), had a healthy body weight (77.4%), identified as middle class (71.7%) and were not in a long-term relationship (54.8%). About 15% of the participants presented problematic alcohol intake, while 26.6% reported a low level of physical activity. Poor sleepers tended to be women, identified as lower class, were smokers, had poor diets and slept fewer hours (*p* < 0.05) (Table 1).

**Figure 2:**
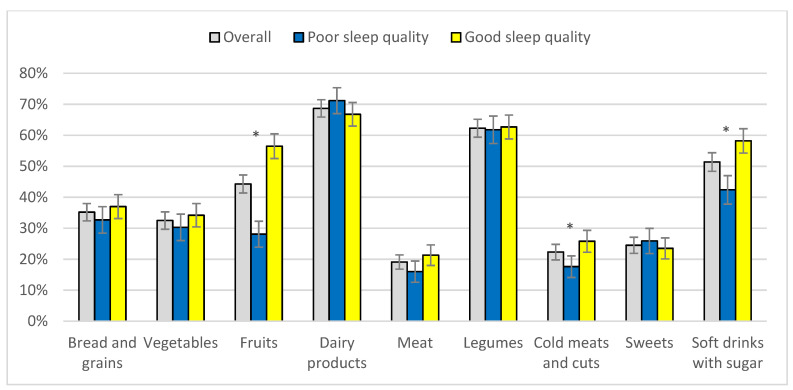
Level of adherence to recommended frequency for eating foods according to sleep quality. * Statistically significant differences between groups (*p* < 0.05 bilateral). Note: Non-adherence to recommended food intake criteria (SENC): bread and grains: less than daily; vegetables: less than daily; fruits: less than daily; dairy products: less than daily; meat: more than twice a week; legumes: less than twice a week; cold meats and cuts: once a week or more; sweets: once a week or more; and soft drinks with sugar: once a week or more.

Most participants’ diets were in need of modification (76.6%), and only 17.7% reported having a healthy diet. The degree to which participants met the SENC dietary recommendations was low in most cases: students did not eat bread and grains, fruits, meat or vegetables with sufficient frequency, whereas they reported the overconsumption of cold meats and cuts, and of sweets (Figure 2).

In total, 51.6% of participants reported poor sleep quality (PSQI score > 5). In the majority of the PSQI subscales, healthy diet was correlated with better sleep quality (i.e., lower PSQI scores). That said, healthy eaters were more likely to use sleep medications (Table 2). Nevertheless, it should be underscored that this difference in the use of medication disappeared when the analysis took the variables of age and gender into account (this information is not reflected in the table).

Binary logistic regression models revealed a strong link between adherence to the SENC dietary recommendations (assessed using the SHEI) and poor sleep quality. Therefore, having a diet in need of modification and, especially, an unhealthy diet was a predictor of poor sleep quality (Table 3).

The same was true for participants who did not adhere to advice concerning the consumption of vegetables, fruits, diary, meat, legumes, sweets, and soft drinks with sugar (Table 4).

## 4. Discussion

A significant percentage of participants reported poor SQ and a diet in need of modification. Furthermore, our results support the hypothesis that poor DQ (i.e., low levels of adherence to the national dietary guidelines for the frequency with which certain foods should be consumed) is significantly associated with poor sleep.

Concerning SQ, just over half of the participants reported suffering from sleep problems (51.6%). This number is noteworthy since it is higher than other figures reported for university students in Norway (30.5%) [46], Spain (30.5%) [46], and Europe more broadly (23.9%) [47]. The prevalence of sleep problems was also higher than that reported in a systematic revision from 2015 [48] that gave a weighted average of 18.5% (95%CI: 11.2–28.8%) for an aggregate sample of 16,478 university students from different countries. This comparison, however, must be viewed with caution given that different methods have been used to analyze data in different studies.

Most study participants reported having a diet that was either unhealthy or in need of modifications (a combined total of 82.2%). These numbers are comparable to other SHEI results for Spanish university students [49,50] as well as the results of parallel indices (also based on Kennedy’s Healthy Eating Index [41]) used to examine university students from other countries [51,52,53,54].

As in the present study, previous research has investigated the connection between diet and sleep through an assessment of adherence to dietary guidelines. A transversal study of Japanese university students (*n* = 175), for instance, examined the link between SQ (also using the PSQI) and DQ, with the latter being determined by adherence to the Japanese Food Guidelines. That study revealed that having a higher-quality diet that was characterized by the balanced intake of meat, fish, eggs, soy products, dairy and fruit was significantly correlated with higher SQ [55]. For their part, Gianfredi et al. [56] studied a group of 117 Italian university students and found that adherence to the Mediterranean diet was linked to increased SQ. The influence of specific food groups has also been observed: an increased intake of animal-based proteins (i.e., meat and fish) was associated with having a greater risk of sleep problems, whereas the consumption of carbohydrates—whether whole or refined grains—was associated with a lower risk of sleep disorders.

These findings appear to be generalizable to all populations. Godos et al. [57] recently carried out a meta-analysis of 29 studies (varied in both research design and target population) in which they concluded that a high-quality diet and the consumption of healthy foods were associated with better SQ, whereas an increased intake of process and sugar-rich foods was linked to worse SQ.

In the present study, participants whose diet did fell short of recommendations for the consumption of vegetables, fruits, dairy, lean meats (including eggs) and legumes tended to have lower SQ; the same was true for those who consumed, in excess, sweets and sugary soft drinks. Such a pattern of not adhering to dietary guidelines—whether due to an excess or lack of consumption—is typical of Western style diets. These diets, which are characterized by high caloric intakes; high levels of refined carbohydrates, animal proteins and saturated fats; and low intakes of fiber and vegetable-derived antioxidants, appear to influence the regulation of the release of glucocorticoids and cortisol through an acidogenic component [58]. Thus, acidotic states with low levels of bicarbonate can stimulate the kidneys to upregulate glutaminase activity and to release cortisol, a situation that plays a leading role in reducing SQ [59]. The association between the release of corticoids and diet ought to be understood as bidirectional given that not only does diet seem to influence hormone release but that unusual levels of glucocorticoids and cortisol could also increase the desire to eat unhealthy foods that are high in calories, fats and free sugars [60,61].

There are other mechanisms that could potentially explain the association between SQ and diet. Irregular sleep could provoke chrononutritional changes and emotional stress and hence an altered decision-making process, which becomes more attuned to seeking out the instant rewards offered by unhealthy foods [62]. Several experimental studies have revealed how partial sleep deprivation leads to increased intakes of fats [63,64], snacks [65,66], foods that are rich in rapidly-absorbed carbohydrates [67,68] and changes in the timing of meals [63]. Similarly, changes in the levels of the appetite hormones ghrelin and leptin, which can be caused by interrupted sleep, can both stimulate desire for foods rich in energy and also reduce desire for vegetables, fruits and legumes [69]. Finally, some foods, such as certain fruits and dairy products, can favor sleep due to their impact on the availability of tryptophan and the synthesis of serotonin and melatonin [70]. All these observed processes support the bidirectional relationship between sleep and diet.

To the best of our knowledge, this is the first study to probe the connection between DQ and sleep in a large group of Spanish university students. There are several reasons that we believe that the present results can be taken as representative of the overall population of Spanish university students: first, the large number of participants in our sample closely reflects the makeup of the larger group terms of age and gender; second, we have used standardized, high-quality tools to collect data; finally, the associations uncovered in the data set are on their face quite plausible and in line with previous findings. The present results provide a reliable profile of the SQ and DQ of university students and support the existence of a meaningful link between these two factors. Despite the study’s strengths, there are nevertheless several limitations that ought to be mentioned. It is possible that the students who perceived their sleep quality to be poor were more inclined to agree to participate in this study. This may have resulted in an overestimation of the prevalence of college students who have a poor sleep quality. Data were self-reported, which means that certain responses could have been biased due to memory lapse or an intentional underreporting of certain aspects, such as the use of sleep medications or body weight [71,72]. For this study, objective means for measuring SQ (e.g., actigraphy or polysomnography) were not employed. Nevertheless, the dimensions of sleep and their importance can shift depending on a participant’s perception of sleep-related issues and daily experience thereof [73]. This is one reason why research methods based on self-reporting are the tools most often used in research. The PSQI, for its part, is one of the most widely used validated tools that allows participants’ to assess their own SQ and has been repeatedly tested and used the world over for studying groups of young adults [74,75].

Concerning the assessment of participants’ diet, the SHEI only examines the frequency with which certain foods are consumed and hence does not measure the amount of food ingested. As a result, it cannot provide information about the intake of nutrients or calories, nor can it provide information about chrononutritional patterns (i.e., regular vs. shifting mealtimes). Despite these real limitations, we have opted to use this tool for various reasons: first, it is a questionnaire that has been specifically adapted to the real dietary habits of Spaniards; second, the SHEI has been repeatedly shown to be quite apt for assessing the DQ of Spaniards [76,77,78]; third, the original version of the questionnaire has been validated with plasmatic biomarkers in previous studies [79,80].

Finally, we must stress that the design of the cross-sectional study allows us to uncover correlations but cannot allow us to determine a strict causal relationship or determine the directionality of the detected associations. Our results may lay the foundations of future studies analyzing the link between diet and SQ. Specifically, prospective, longitudinal studies in this area should address the biological paths relating intake of specific foods and sleep. In addition, future experimental studies should analyze the impact of specific diet and food intake prescription on SQ.

## 5. Conclusions

The purpose of the present study was to evaluate the link between diet and SQ in a large sample of Spanish university students. The data analysis has underscored three main findings: first, sleeping disorders are common among Spanish university students, as shown by the fact that more than half of participants reported poor SQ; second, Spanish university students’ DQ is, on the whole, poor, with 82.2% of participants reporting diets that required some degree of modification; third, there is a strong correlation between dietary habits and SQ. Thus, the underconsumption of certain foods (vegetables, fruits, diary, lean meats, and legumes) as well as the overconsumption of others (sugary soft drinks and sweets) were both associated with a higher risk of suffering from a sleeping disorder.

These findings signal the importance of implementing new health-related policies that can both lead to future research and promote healthy sleeping habits among university students. Given the association between SQ and DQ, any sleep exam should also contain an analysis of the patient’s diet. Given that we find it completely plausible that the relation between sleep and diet is bidirectional, we support any actions directed to breaking the vicious cycle in which diet can reduce SQ, and sleeping disorders can, in turn, lead to a reduction in DQ.

## Figures and Tables

**Table 1:** Characteristics of the study population according to their sleep quality.

Variables	Total *n* = 868 (100%)	Good Sleep Quality *n* = 420 (48.4%)	Poor Sleep Quality *n* = 448 (51.6%)	*p*
Age	22.84 ± 7.50	23.05 ± 7.89	22.64 ± 7.12	0.148
Sex				
Male	189 (21.8%)	119 (63.0%)	70 (37.0%)	0.000
Female	679 (78.2%)	301 (44.3%)	378 (55.7%)	
BMI (kg/m^2^)	22.20 ± 3.18	21.94 ± 2.93	22.44 ± 3.38	0.015
BMI categories				
Under weight (<18.5 kg/m^2^)	84 (9.7%)	35 (41.7%)	49 (58.3%)	
Normal weight (18.5–24.9 kg/m^2^)	672 (77.4%)	343 (51.0%)	329 (49.0%)	0.005
Over-weight (25–29.9 kg/m^2^)	70 (8.1%)	21 (30.0%)	49 (70.0%)	
Obese (≥30 kg/m^2^)	42 (4.8%)	21 (50.0%)	21 (50.0%)	
Relationship status				
Without a stable partner	476 (54.8%)	224 (47.1%)	252 (52.9%)	0.413
With a stable partner	392 (45.2%)	196 (50.0%)	196 (50.0%)	
Grade point average (min. 0–max. 10)	7.14 ± 0.62	7.15 ± 0.65	7.13 ± 0.59	0.931
Perceived social class				
Lower	210 (24.2%)	77 (36.7%)	133 (63.3%)	0.000
Middle	623 (71.7%)	322 (51.7%)	301 (48.3%)	
Upper	35 (4.0%)	21 (60.0%)	14 (40.0%)	
Smoking habits				
Smoker	280 (32.3%)	98 (35.0%)	182 (65.0%)	0.000
Non-smoker	588 (67.7%)	322 (54.8%)	266 (45.2%)	
CAGE Score	0.62 ± 0.91	0.58 ± 0.91	0.66 ± 0.90	0.062
CAGE categories				
Non-risky alcohol consumption	742 (85.5%)	364 (49.1%)	378 (50.9%)	0.386
Risky alcohol consumption	126 (14.5%)	56 (44.4%)	70 (55.6%)	
Screen time/day	3.40 ± 1.72	3.53 ± 1.77	3.27 ± 1.65	0.097
Physical activity				
Low	231 (26.6%)	105 (45.5%)	126 (54.5%)	0.076
Medium	371 (42.7%)	196 (52.8%)	175 (47.2%)	
High	266 (30.6%)	119 (44.7%)	147 (55.3%)	
SHEI Score	69.88 ± 11.28	71.43 ± 11.56	68.44 ± 10.83	0.000
SHEI categories				
Unhealthy diet	49 (5.6%)	21 (42.9%)	28 (57.1%)	0.000
Diet needing modifications	665 (76.6%)	294 (44.2%)	371 (55.8%)	
Healthy diet	154 (17.7%)	105 (68.2%)	49 (31.8%)	
Hours of sleep/day	7.15 ± 1.19	7.65 ± 1.26	6.68 ± 0.89	0.000
PSQI total (min. 0–max. 21)	6.39 ± 3.58	3.52 ± 1.26	9.08 ± 2.89	0.000
PSQI dimensions				
Subjective sleep quality (min. 0–max. 3)	0.98 ± 0.82	0.45 ± 0.49	1.47 ± 0.77	0.000
Sleep latency (min. 0–max. 3)	1.43 ± 1.00	0.67 ± 0.59	2.14 ± 0.74	0.000
Sleep duration (min. 0–max. 3)	0.74 ± 0.74	0.33 ± 0.53	1.13 ± 0.69	0.000
Habitual sleep efficiency (min. 0–max. 3)	0.49 ± 0.76	0.10 ± 0.30	0.86 ± 0.88	0.000
Sleep disturbances (min. 0–max. 3)	1.13 ± 0.47	0.97 ± 0.36	1.28 ± 0.51	0.000
Use of sleep medications (min. 0–max. 3)	0.28 ± 0.71	0.05 ± 0.21	0.50 ± 0.92	0.000
Daytime dysfunction (min. 0–max. 3)	1.34 ± 0.82	0.95 ± 0.61	1.70 ± 0.82	0.000

**Table 2:** PSQI scores based on diet quality.

	Unhealthy Diet (a)	Needed Changes Diet (b)	Healthy Diet (c)	*p*	Post-Hoc Pairwise Comparisons
Subjective sleep quality (min. 0–max. 3)	1.29 ± 0.70	1.00 ± 0.83	0.77 ± 0.79	0.000	a, b > c
Sleep latency (min. 0–max. 3)	1.71 ± 1.17	1.46 ± 0.93	1.18 ± 1.16	0.001	a, b > c
Sleep duration (min. 0–max. 3)	0.43 ± 0.50	0.81 ± 0.77	0.55 ± 0.58	0.000	b > a, c
Habitual sleep efficiency (min. 0–max. 3)	0.43 ± 0.74	0.54 ± 0.81	0.32 ± 0.56	0.011	b > c
Sleep disturbances (min. 0–max. 3)	1.00 ± 0.54	1.15 ± 0.48	1.09 ± 0.42	0.093	--------
Use of sleep medications (min. 0–max. 3)	0.14 ± 0.35	0.24 ± 0.63	0.50 ± 1.04	0.040	c > b
Daytime dysfunction (min. 0–max. 3)	1.57 ± 0.74	1.36 ± 0.80	1.18 ± 0.83	0.005	a, b > c
PSQI total (min. 0–max. 21)	6.57 ± 3.83	6.56 ± 3.61	5.59 ± 3.25	0.003	b > c

**Table 3:** Relationship between diet quality and presence of poor sleep quality (PSQI score > 5).

Independent Variable	Unadjusted OR (CI 95%)	Adjusted OR (CI 95%) *
Healthy diet	Reference	Reference
Diet needing modifications	2.704 (1.864, 3.923) **	2.791 (1.874, 4.157) **
Unhealthy diet	2.857 (1.477, 5.526) **	4.201 (2.071, 8.525) **

* Adjusted for age (continuous), gender (male/female), physical activity (low/medium/high), perceived social class (lower/middle/upper), BMI (<18.5 kg/m^2^/18.5–24.9 kg/m^2^/≥25 kg/m^2^), smoker (yes/no), problematic alcohol use (yes/no), academic performance (continuous), relationship status (yes/no) and screen time (continuous). ** *p* < 0.005.

**Table 4:** Relationship between not adhering to national guidelines for consuming different foods and the presence of poor sleep quality (PSQI score > 5).

Independent Variable	Unadjusted OR (CI 95%)	Adjusted OR (CI 95%) *
Bread and grains ^a^	0.933 (0.682, 1.277)	0.872 (0.618, 1.230)
Vegetables ^a^	1.591 (1.147, 2.206) **	1.637 (1.143, 2.345) **
Fruit ^a^	3.761 (2.771, 5.104) **	4.085 (2.906, 5.742) **
Dairy ^a^	1.681 (1.241, 2.276) **	1.964 (1.417, 2.723) **
Meat (including eggs) ^a^	1.581 (1.104, 2.265) **	1.826 (1.196, 2.786) **
Legumes ^a^	1.120 (0.823, 1.525)	1.430 (1.009, 2.027) **
Cold meats and cuts ^b^	0.774 (0.544, 1.101)	0.764 (0.525, 1.111)
Sweets ^b^	1.776 (1.282, 2.460) **	1.603 (1.138, 2.257) **
Soft drinks with sugar ^b^	1.747 (1.312, 2.326) **	1.462 (1.071, 1.995) **

^a^ Category that was underconsumed. ^b^ Category that was overconsumed. * Adjusted for age (continuous), gender (male/female), physical activity (low/medium/high), perceived social class (lower/middle/upper), BMI (<18.5 kg/m^2^/18.5–24.9 kg/m^2^/≥25 kg/m^2^), smoker (yes/no), problematic alcohol use (yes/no), academic performance (continuous), relationship status (yes/no) and screen time (continuous). ** *p* < 0.005.

## Data Availability

The data presented in this study are available on request from the corresponding author. The data are not publicly available as they contain information that could compromise the privacy of research participants.

## Supplementary Materials

The following supporting information can be downloaded at: https://www.mdpi.com/article/10.3390/nu14163291/s1, Table S1: Criteria for scoring the Spanish Healthy Eating Index (SHEI) [42,43].
