# Supplementary material for: The Association between Diet and Sleep Quality among Spanish University Students
Source: Nutrients. 2022 Aug 11;14(16):3291. doi: 10.3390/nu14163291 (PMC9415359; doi:10.3390/nu14163291)
Supplement: Supplementary file 1 [file nutrients-14-03291-s001.zip › nutrients-1873099-supplementary.pdf]

Supplementary Table S1. Criteria for scoring the Spanish Healthy Eating Index (SHEI)[1].

Category	Criteria for a score of 10*	Criteria for a score of 7.5	Criteria for a score of 5	Criteria for a score of 2.5	Criteria for a score of 0
**Bread and grains**	Consumed daily	3 or more times a week but not daily	Once or twice a week	Less than once a week	Never or very rarely
**Vegetables**	Consumed daily	3 or more times a week but not daily	Once or twice a week	Less than once a week	Never or very rarely
**Fruits**	Consumed daily	3 or more times a week but not daily	Once or twice a week	Less than once a week	Never or very rarely
**Dairy products**	Consumed daily	3 or more times a week but not daily	Once or twice a week	Less than once a week	Never or very rarely
**Meat**	Once or twice a week	3 or more times a week but not daily	Less than once a week	Consumed daily	Never or very rarely
**Legumes**	Once or twice a week	3 or more times a week but not daily	Less than once a week	Consumed daily	Never or very rarely
**Cold meats and cuts**	Never or very rarely	Less than once a week	Once or twice a week	3 or more times a week but not daily	Consumed daily
**Sweets**	Never or very rarely	Less than once a week	Once or twice a week	3 or more times a week but not daily	Consumed daily
**Soft drinks with sugar**	Never or very rarely	Less than once a week	Once or twice a week	3 or more times a week but not daily	Consumed daily
**Variety of diet**	1 point if the participant completes each of the weekly recommendations and two points if the participant each of the daily recommendations.				

*A score of 10 in a particular category means that the participant's habits are aligned with the frequency recommendations for food consumption provided by the Spanish Society for Community Nutrition[2].

References

(1) Norte Navarro, A. I.; Ortiz Moncada, R. [Spanish diet quality according to the healthy eating index]. *Nutr. Hosp.* **2011**, *26* (2), 330–336. https://doi.org/10.1590/S0212-16112011000200014.

(2) Grupo Colaborativo de la Sociedad Española de Nutrición Comunitaria (SENC; Aranceta Bartrina, J.; Arija Val, V.; Maíz Aldalur, E.; Martínez de la Victoria Muñoz, E.; Ortega Anta, R. M.; Pérez-Rodrigo, C.; Quiles Izquierdo, J.; Rodríguez Martín, A.; Román Viñas, B.; Salvador Castell, G.; Tur Marí, J. A.; Varela Moreiras, G.; Serra Majem, L. Dietary guidelines for the Spanish population (SENC, December 2016); the new graphic icon of healthy nutrition. *Nutr. Hosp.* **2016**, *33* (Suppl 8), 1–48. https://doi.org/10.20960/nh.827.
